# Commentary: Correlation of prefrontal cortical activation with changing vehicle speeds in actual driving: a vector-based functional near-infrared spectroscopy study

**DOI:** 10.3389/fnhum.2015.00665

**Published:** 2015-12-09

**Authors:** Noman Naseer

**Affiliations:** Department of Mechatronics Engineering, Air UniversityIslamabad, Pakistan

**Keywords:** fNIRS, BCI, vector-based phase analysis, hemodynamic response, prefrontal cortex

I am really impressed by this article published in Frontiers in Human Neuroscience in December 2013. In this paper, Yoshino et al. (2013) demonstrate that the activation of prefrontal cortex increases with faster deceleration during driving, which implies that during a quick brake the driver's brain-functionality increases which is required to avoid accidents. While the above mentioned finding of this study is of interest to many, the real strength of this paper, in my opinion, lies in the use of vector-based phase analysis of fNIRS signals.

The vector-based method appears to be a powerful method to analyze fNIRS signals. Examination of several indices including the changes in the concentration of oxygenated hemoglobin (ΔHbO), deoxygenated hemoglobin (ΔHbR), cerebral blood volume (ΔCBV), cerebral oxygen exchange (ΔCOE), activity strength (*L*), and the phase angle (*k*), simultaneously on a single plot, might yield to new insights of the fNIRS data and its relationship with neuronal activity. One of the major advantages of the vector-phase diagram is that it can be plotted for each data point in real-time which make it very easy for us to visualize the relationship between the fNIRS indices in real-time. The position of the vector on the vector-phase diagram can easily show the degree of oxygen exchange and the oxygen demand.

The use of vector-based phase analysis of the fNIRS signals might yield to interesting results in fNIRS-based brain-computer interfaces (BCI). For BCI purposes classification of features is required to generate control commands (Naseer and Hong, 2015): Several novel features including different combinations of *L, k*, ΔCBV, and ΔCOE can be extracted, and used for classification, using the vector-based phase analysis. These features might add a new dimension to conventional features currently being used in classification for BCI purposes. For example, Naseer and Hong (2013) used signal slope (SS) and signal mean (SM) values of ΔHbO and ΔHbR, calculated during a smaller time span of 2–7 s within the 10 s task period as the features for classification. Using linear discriminant analysis for classification, they attained classification accuracies of up to 87% to discriminate between SS and SM values of two different mental tasks. It would be interesting to see the classification results acquired using *L, k*, CBV, and COE values acquired over the same period of time. The classification results, in my opinion, are expected to be better than those achieved in the previous study since *L* and *k* directly represent the degree of oxygen exchange and oxygen demand, which characterize the neuronal firing in a specific brain area. Further research needs to be carried out using vector-phase-based features to establish proposed improvement and the reliability of fNIRS-based BCI systems.

## Conflict of interest statement

The author declares that the research was conducted in the absence of any commercial or financial relationships that could be construed as a potential conflict of interest.
